# Transcriptomic and protein–protein interaction network analyses of the molecular mechanisms underlying the mycorrhizal interaction in *Cypripedium macranthos* var. *rebunense*

**DOI:** 10.3389/fpls.2025.1597154

**Published:** 2025-07-10

**Authors:** Chunyi Jin, Kota Kambara, Kaien Fujino, Hanako Shimura

**Affiliations:** ^1^ Faculty of Agriculture, Hokkaido University, Sapporo, Japan; ^2^ Asian Research Center for Bioresource and Environmental Sciences (ARC-BRES), Graduate School of Agricultural and Life Sciences, The University of Tokyo, Tokyo, Japan

**Keywords:** *Cypripedium macranthos*, *Tulasnella* sp., mycorrhizal fungi, RNA-seq, transcriptome analysis

## Abstract

**Introduction:**

Orchid mycorrhizal (OM) symbiosis plays an essential role in orchid seed germination and development, but its molecular mechanisms remain largely unexplored.

**Methods:**

To comprehensively analyze gene expression associated with early fungal colonization, transcriptome analysis of *Cypripedium macranthos* var. rebunense was performed using mycorrhizal tissues prepared by inoculating seedling plants with a fungus that exhibited different mycorrhizal interaction properties among subcultures.

**Results:**

Colonization with the mycorrhizal fungus induced an increased expression of orchid genes encoding enzymes involved in cell wall synthesis, degradation, and modification, as well as those encoding transporters of sugars, amino acids, nucleic acids, and other nitrogen-containing compounds. Enrichment analysis focusing on genes associated with protein–protein interactions (PPI) suggested a potential role of lectin domain-containing receptor-like kinases (LecRLKs) in the recognition of fungal colonization and the induction of cell wall-modifying enzymes and nutrient transporters required for mycorrhizal formation. Kinase genes such as MAPKKK and serine/threonine protein kinase were upregulated in tissues exhibiting continued peloton formation, whereas these genes exhibited no changes in tissues showing no peloton formation four weeks after inoculation.

**Discussion:**

These results suggest that the continuous phosphorylation signaling cascade plays a crucial role in the regulatory pathway for maintaining mycorrhizal interactions between *Cypripedium* and its mycorrhizal fungus.

## Introduction

The family Orchidaceae presents one of the most diverse groups of angiosperms, with approximately 28,000 species distributed worldwide across various ecological environments (Chase et al., 2015). Unlike most seed plants, orchid seeds are extremely small and referred to as “dust-like seeds”; they lack an endosperm, and the immature embryo is enclosed in a tough seed coat that prevents autonomous germination (Pujasatria et al., 2020; Bidartondo and Read, 2008; Lee and Yeung, 2023). Under natural conditions, orchid seeds must establish a relationship with orchid mycorrhizal fungi (OM fungi) belonging to basidiomycetes or ascomycetes to obtain essential nutrients, such as carbon and nitrogen compounds, required for the germination and growth of juvenile seedlings (McCormick et al., 2018; Rasmussen et al., 2015). The important features of interactions between orchid and OM fungi include intracellular colonization by OM fungal hyphae in orchid cells, forming tightly coiled structures known as pelotons. Pelotons are surrounded by the host plasma membrane that provides a specific interface for nutrient exchange (Perotto and Balestrini, 2024; Leng et al., 2024). As the mycorrhizal relationship progresses, these peloton structures eventually degrade, followed by the formation of new pelotons presenting a cyclic process (González-Chávez et al., 2018; Li et al., 2020).

In the mycorrhizal relationship between orchids and OM fungi, the dependence of orchids on fungi for seed germination makes orchids a unique case in diverse mycorrhizal relationships. Although the orchid–OM fungal interactions have unique characteristics regarding nutrient transfer between the two organisms, the regulatory mechanism underlying the OM interactions has similarities with other mycorrhizal associations such as arbuscular mycorrhiza (AM) (Zhao et al., 2014; Favre-Godal et al., 2020; Genre et al., 2020; Reiter et al., 2020); common symbiotic genes (CSGs) induced in AM relationship were also upregulated in orchid–OM fungi interaction (Miura et al., 2018). In recent years, several molecular mechanisms underlying OM interactions have been reported. For instance, the importance of sugar transport in orchid–OM fungi interactions was reported; *Sugars will eventually be exported transporters* (*SWEET*) genes (sugar transporters) are significantly induced during orchid seed germination in the presence of fungi (Zhao et al., 2024). Additionally, nitrogen metabolism plays a crucial role in the interaction; Fochi et al. suggest that OM fungi take up ammonium (NH^4+^) from the interface between orchid and fungal cells via fungal ammonium transporters, and the orchid obtains amino acids or oligopeptides from the fungus (Fochi et al., 2017). The involvement of active defense responses in the OM interaction varies among species; while a few studies indicated that orchids establish a relatively non-defensive, “friendly relationship” with their fungal partners, others have reported that certain orchid species activate host defense genes during OM interaction (Perotto et al., 2014; Zeng et al., 2018; Yu et al., 2023). Moreover, OM interactions may involve hypoxic responses (Xu et al., 2023); however, the regulatory mechanisms associated with these processes remain unclear.

Despite recent efforts to explore the functional aspects associated with the OM relationship, the molecular mechanisms involved remain less understood compared to the AM and ectomycorrhiza (ECM) interactions. Furthermore, of the more than 700 genera in the Orchidaceae family, genome information is currently available for only 12, including *Dendrobium*, *Phalaenopsis*, *Apostasia*, *Platanthera*, and *Ophrys*, which still poses challenges for deciphering the molecular regulatory networks underlying OM interactions (Cai et al., 2015; Zhang et al., 2016, 2017; Li et al., 2022; Russo et al., 2024). For species with unidentified genome sequences, gene-coding sequences can be obtained *de novo* from RNA sequencing (RNA-seq) data and then analyzed for comprehensive gene expression profiles (transcriptomes). In orchid species, RNA-seq analysis has been used to explore the molecular mechanisms involved in various physiological and biological events, such as floral organ formation, synthesis of secondary metabolites, and interactions with microbes (Ahmad et al., 2022; Liu et al., 2022; Pujasatria et al., 2024). In our previous report, the RNA-seq analysis of *Cypripedium macranthos* var. *rebunense*, an endangered lady slipper orchid in Japan, during its seed germination with the OM fungus, revealed the induction of CSGs and defense-related genes (Kambara et al., 2023). However, because of limitations in sample replication, the results were not subjected to sufficient statistical evaluation.

In this study, we used alternative materials in which the OM fungus was inoculated to *in vitro* seedlings to obtain sufficient material for RNA-seq and identify significant changes in gene expression related to early fungal colonization by OM fungi. We here used two subcultures of the OM fungus WO97 (Shimura et al., 2009), both belonging to the genus *Tulasnella* and sharing identical rDNA-ITS sequences. Despite being vegetatively propagated clones, these subcultures have shown differing symbiotic performances: one (WO97-R) consistently induced protocorm formation, while the other (WO97-N) exhibited variable or unstable colonization in some replicates (our unpublished data). To examine how the orchid host responds to fungal partners with different colonization capabilities, we performed RNA-seq using root tissues inoculated with either subculture.

There are still few examples of genetic analysis in the Orchidaceae family, and the limited annotations do not fully explain the broader mechanisms underlying OM interactions; therefore, we applied STRING-based protein–protein interaction (PPI) network analysis to identify key genes involved in cellular signaling events. The STRING database is a powerful tool for predicting PPI networks by integrating experimentally validated interactions, co-expression analyses, protein structure predictions, and computationally inferred associations (Szklarczyk et al., 2023). STRING-based PPI networks have been increasingly adopted in plant research to facilitate the prediction of downstream targets of TFs, identify hub proteins, and explore regulatory networks (Valencia-Lozano et al., 2022; Wimalagunasekara et al., 2023; Yu et al., 2024). By integrating differential gene expression analysis with PPI network prediction, we aimed to uncover core regulatory pathways that reflect the host’s response to variable fungal colonization.

## Materials and methods

### Plant and fungal materials

The experimental setup for preparing materials for RNA-seq is shown in **Supplementary Figure S1A**. Considering the challenges in collecting sufficient symbiotically germinated protocorm samples for transcriptome analyses, we cultured the symbiotically germinated protocorms and grew them. The resulting seedlings that developed roots were used as plant material for this study. First, to prepare protocorms by symbiotic germination, mature capsules of *C. macranthos* var. *rebunense* were collected from Rebun Island, Japan in September 2021, with permission from the Ministry of Environment, Japan. *In vitro* symbiotic germination was conducted as described previously using the OM fungus WO97, isolated from the roots of *C*. *macranthos* var. *rebunense* (Shimura et al., 2009; Kambara et al., 2023). We here used two subcultures of the OM fungus WO97, WO97-R and WO97-N. These two are vegetatively propagated clones derived from WO97, and their rDNA-ITS sequences are identical. These subcultures have been maintained in our laboratory and used in annual germination assays for *C. macranthos* var. *rebunense*. In preliminary experiments in symbiotic germination, we have observed that the ability of the WO97 strain to induce germination varied depending on the subcultured mycelium: WO97-R, which induced seed germination and protocorm development *in vitro* without differences between replicates and exhibited a stable symbiotic relationship, and WO97-N, which induced germination and protocorm growth at frequencies varying among replicates and exhibited an unstable relationship. To examine whether there were differences in the responses of the orchid when inoculated by subcultures with different colonization capabilities, WO97-R and WO97-N were selected in this experiment.

After approximately 6 months of co-cultivation with the fungus, well-grown protocorms with meristem formation were transferred to a modified T medium (based on Tsutsui and Tomita, 1990, with yeast extract excluded) supplemented with a fungicide (Tsutsui and Tomita, 1990; Shimura and Koda, 2005). Protocorms generated by inoculation with WO97-R and those by inoculation with WO97-N were transplanted separately (**Supplementary Figure S1A**). An incubation step with a fungicide was performed to promote protocorm growth and root development on a nutrient-containing medium. The step was also performed to eliminate fungal hyphae that had colonized the orchid tissues during germination, prior to the second inoculation. To prepare the inoculum plate for the second inoculation, a piece of agar containing WO97 hyphae was excised from the plate on which the protocorms had been transplanted; subsequently, the hyphae were allowed to grow again on fresh oatmeal medium and used for the second inoculation. The 20–30 seedlings cultured with fungicide for 4 months were transferred to the WO97-R or WO97-N growing oatmeal medium and further cultured at 20°C for 2 or 4 weeks.

To evaluate fungal colonization, a piece of tissue was taken from each of a few root slices prepared for RNA extraction, fixed in formalin–acetic acid–alcohol (FAA), and 10 to 11 sections from each inoculated seedling were used for observation. Fungal colonization and peloton formation were observed according to Yamamoto et al. (2017). This method has been used to observe protocorm tissue infected with fungi (Yamamoto et al., 2017), but it was also applicable to the mycorrhizal tissue of the small seedlings’ roots used in our experiment. After removing the fibrous tissues using a dissecting needle, the stained root tissues were transferred onto a microscope slide. Then, the colonization status of the cells was observed under a microscope (Nikon ECIPSE E600, Tokyo, Japan), and images were captured using a Penguin 600CL (Pixera, Osaka, Japan).

### RNA sequencing

For RNA extraction, root tissue slices (approximately 0.05 g) were collected from 8–10 seedlings after 2 and 4 weeks of culture; each group of samples included three replicates. Root tissues from seedlings before fungal inoculation (pre-inoculation) were prepared in triplicates as controls. Total RNA was extracted and treated with DNase following a previously described protocol (Shimura et al., 2018), and the purified RNA was used in RNA-seq. Library preparation using the TruSeq Stranded mRNA Sample Prep Kit (Illumina) and sequencing on the Illumina NovaSeq platform were outsourced to Macrogen Inc. (Seoul, Republic of Korea). Each library was sequenced, yielding approximately 50 million 151-bp paired-end reads. Data obtained by RNA-seq were submitted to the National Center for Biotechnology Information (NCBI) Sequence Read Archive (SRA) database (PRJNA1231755).

### Pre-processing and *de novo* assembly

The raw reads were processed using fastp v0.23.4 (Chen, 2023) for base correction, adapter trimming, and quality filtering. Considering the unavailability of the *C. macranthos* var. *rebunense* genome sequence, reference sequences were constructed by *de novo* assembly using Trinity v2.15.1 (Grabherr et al., 2013). To expedite the assembly process, one-third of the read data (ca. 1.66 Gb) were randomly extracted from each of the three replicates in a treatment and merged using seqkit v2.1.0 (Shen et al., 2016) to create representative read data for each treatment. Five representative reads were used as input data for *de novo* assembly on Trinity. Redundant contigs were removed from the assembly using CD-HIT-EST v4.8.1 (parameter -c set to 0.8, Li and Godzik, 2006) and the EvidentialGene tr2aacds pipeline (Gilbert, 2013; http://arthropods.eugenes.org/EvidentialGene/about/EvidentialGene_trassembly_pipe.html). The quality of the contig set was assessed based on the contig number, mapping rate, and BUSCO (v5.6.1) completeness, following a previous report (Kambara et al., 2023).

To determine the effects of the fungus-derived reads or contigs on subsequent analyses, we prepared a contig set that excluded as many fungal sequences as possible. We used hisat2 v2.2.1 (Kim et al., 2019) to identify the reads that were significantly mapped to the WO97 draft genome sequence (our unpublished data). The mapped reads were removed from each sample’s clean reads using samtools v1.6 (Li et al., 2009), and *de novo* assembly was conducted using the remaining reads as described above. WO97-related contigs were further identified in the assembled contigs. Specifically, BLASTn alignment against the contig set was performed by setting the predicted CDS sequences from the WO97 draft genome as a reference and stringent parameters (e-value < 1e-50, identity ≥ 90%). Identified hit contigs were considered WO97-derived transcripts, and the corresponding contigs were removed from the contig set as described in the Results section.

### Differentially expressed gene analysis and functional annotation

The abundance of the transcripts was estimated by mapping the total clean reads to the contig set using Bowtie2 v2.5.3 (Haas et al., 2013), converted to BAM format using samtools v1.19.2 (Cock et al., 2015), and quantified by salmon v1.12.0 (Patro et al., 2017). For differentially expressed gene (DEG) analysis, contig sets without WO97-related sequences were used for mapping, because fungal transcripts in mycorrhizal tissues would certainly be detected as DEGs when comparing with non-mycorrhizal tissues. DEGs were identified using the DESeq2 package (R, v4.3.3) considering a log2 fold change (FC) threshold > |1| and an adjusted *p*-value (*p*_adj_) < 0.05. The amino acid sequences of the contigs were annotated using DIAMOND BLASTP v2.1.9.163 (Buchfink et al., 2021) against the RefSeq plant database (O’Leary et al., 2016). Functional annotations were further refined using InterProScan v1.8.0 (Jones et al., 2014) for Gene Ontology (GO) term assignment and KofamKOALA v1.3.0 (Aramaki et al., 2020) for the Kyoto Encyclopedia of Genes and Genomes (KEGG) Orthology profile. For KEGG pathway identification, the most relevant pathway ID for each K number was retained based on its frequency or the highest association score. Venn analysis was performed using the online tool Jvenn (https://www.bioinformatics.com.cn/static/others/jvenn/, Bardou et al., 2014).

### Enrichment analysis and PPI network establishment

GO and KEGG pathway enrichment analyses were performed using R v4.3.3 with the clusterProfiler and ggplot package. GO and KEGG enrichment significance was assessed using BH multiple testing correction, with a threshold of *p*_adj_ ≤ 0.05 to define significantly enriched GO terms and KEGG pathways. The heatmap was generated based on the Python 3.9, Seaborn v0.12.2, and Matplotlib v3.7.2 libraries. PPI networks were constructed with the STRING platform (https://string-db.org/) using *Dendrobium catenatum* as the reference species. Proteins with sequence identities below 30% were excluded to ensure the accuracy of the analysis. When we uploaded our DEG sequences to STRING, only two orchid species (*D. catenatum* and *Apostasia shenzhenica*) returned as matched proteins. Among these, *D. catenatum* yielded more matched sequences, allowing for broader gene coverage in the network analysis. Therefore, we chose *D. catenatum* as the reference species to ensure more comprehensive and reliable interaction predictions. The *SWEET* genes are incorporated as anchor genes to prioritize mycorrhizal interaction-related genes, minimizing the inclusion of irrelevant genes. The confidence threshold was set at 0.2, which presents the minimum level required to include the *SWEET* genes in the network while maintaining a manageable scale. Unconnected nodes were excluded to enhance visualization and to focus on biologically meaningful interactions. To calculate the node weights, the refined network was further analyzed using Cytoscape v3.10.3 (Shannon et al., 2003) based on dual centrality metrics [betweenness centrality (BC) and degree centrality]. Genes ranked within the top 30% for both metrics were classified as key genes, which represented the central regulatory hubs of DEGs.

### Statistical analysis

The significance of the differences in the proportion of fungal reads at different times after inoculation [2 weeks post inoculation (wpi) and 4 wpi] was evaluated using Tukey–Kramer multiple comparison. DEG detection and GO and KEGG enrichment analyses were conducted with a threshold of *p*_adj_ ≤ 0.05 to define significant genes. All statistical analyses were conducted in R v4.3.3.

## Results

### Assessment of colonization in *Cypripedium macranthos* var. *rebunense* seedlings by OM fungus

**Figure 1:**
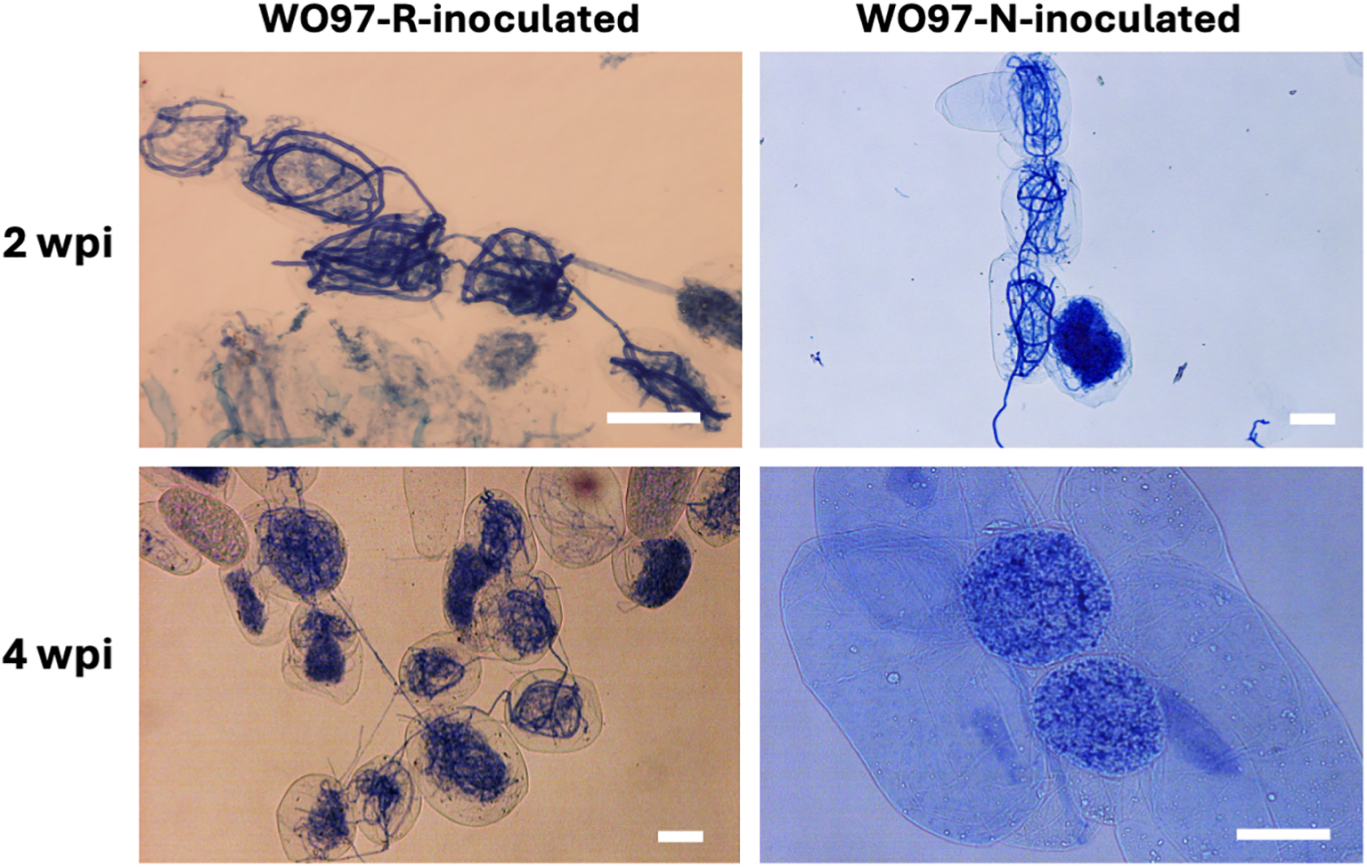
Hyphal coils formed in root cells of *Cypripedium macranthos* var. *rebunense* seedlings. *In vitro* grown seedlings of *C. macranthos* var. *rebunense* were inoculated with a mycorrhizal fungus (WO97-R or WO97-N), and then hyphal coil formation (peloton formation) in the root tissues was observed 2 and 4 weeks post inoculation (wpi). Scale bars, 50 μm.

At both sampling time points, the seedlings in the WO97-R and WO97-N treatments had well-developed roots and were similar in overall morphology (**Supplementary Figure S1B**). There was no visible difference in growth compared to the control (pre-inoculation) during the incubation period. To evaluate the colonization and subsequent mycorrhizal development, we observed peloton formation inside the root tissues of *C. macranthos* var. *rebunense* seedlings at 2 and 4 wpi. In the control root tissue of the seedlings before inoculation, no peloton formation was observed. At 2 wpi, the hyphae were distinctly visible and colonized in cells, and peloton formation was induced by both WO97 cultures in orchid root cells (**Figure 1**; **Supplementary Figure S2A**). At 2 wpi, there was no clear difference in the number of cells that were confirmed to be colonized with hyphae between WO97-R-inoculated and WO97-N-inoculated tissues. However, in the WO97-N-inoculated root tissues, few elongated hyphae were observed at 4 wpi, and the pelotons showed structural breakdown and light staining, indicating potential hyphal degradation. On the other hand, elongated hyphae and peloton formation were observed at 4 wpi in the root tissue inoculated with WO97-R, as observed at 2 wpi (**Figure 1**; **Supplementary Figure S2A**).

### RNA-seq of inoculated *C. macranthos* var. *rebunense* seedlings

**Figure 2:**
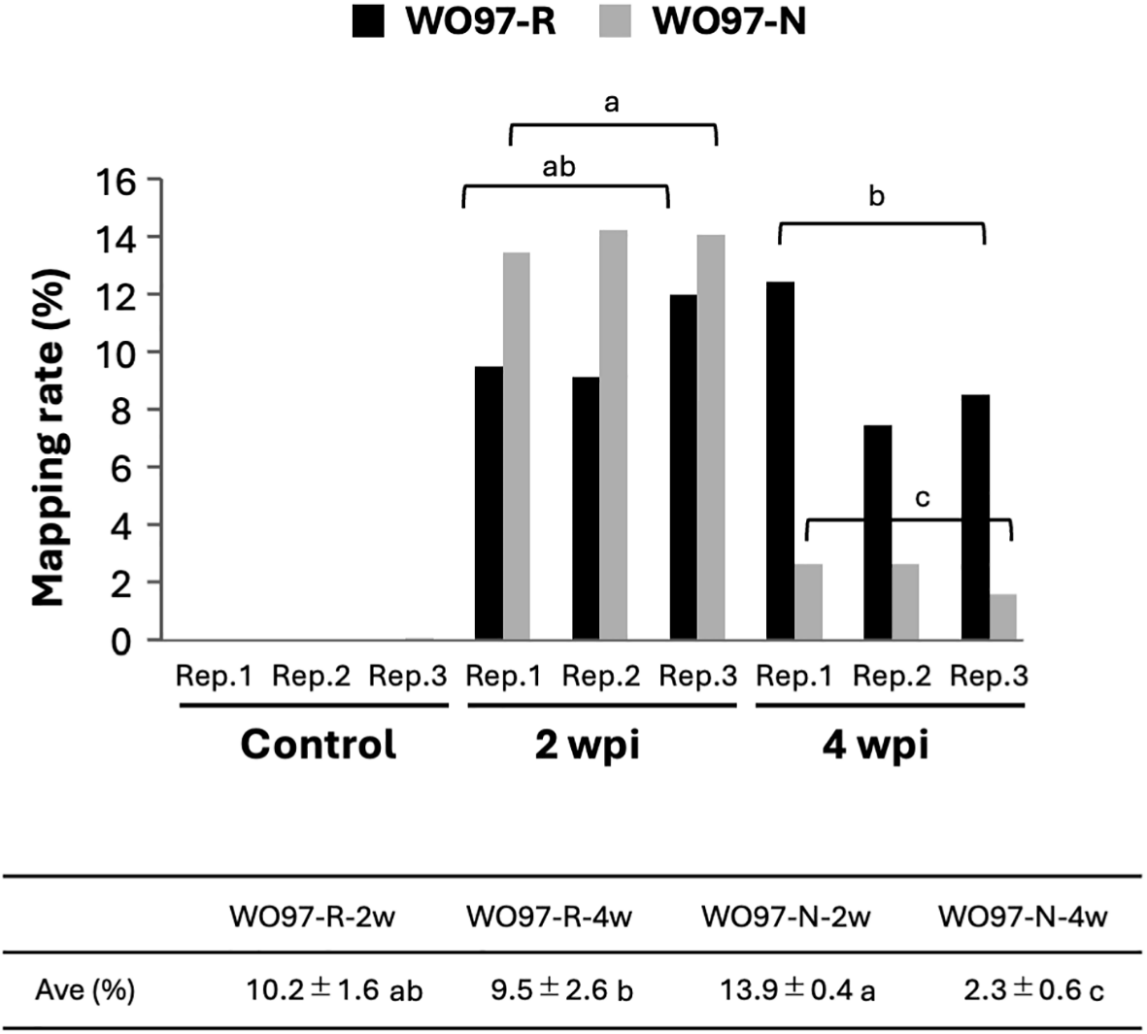
Quantitative analysis of fungi-derived short reads in RNA-seq data. Among the total clean reads, short reads mapped to the WO97 draft genome sequence were considered fungi-derived reads, and the percentages were estimated. Each experiment included three biological replicates. The control group represented conditions with no fungal inoculation. The table shows the average fungal reads percentages in the inoculated samples. The significance of the differences in the proportion of fungi-derived short reads at different times after inoculation (2 wpi and 4 wpi) was evaluated using Tukey–Kramer multiple comparison; values with different lowercase letters are significantly different at *p* < 0.01.

RNA-seq of 15 samples from five treatment groups (Control, WO97-R-2w, WO97-R-4w, WO97-N-2w, and WO97-N-4w) yielded 93.5%–94.2% of clean reads with a quality score of Q30 (sequencing error rate < 0.1%) from total reads. To examine the abundance of WO97-derived transcripts in each sample, we identified the short reads mapped to the WO97 draft genome sequence as WO97-derived transcripts and calculated their proportions. In the control sample, 0.0% of the reads presented WO97-derived transcripts, whereas the percentage of mapped reads was as high as 14% of the total clean reads in the inoculated treatments (WO97-R-2w, WO97-R-4w, WO97-N-2w, and WO97-N-4w) (**Figure 2**). In WO97-R-inoculated tissues, the proportion of WO97-derived reads did not significantly differ between 2 and 4 wpi; however, in WO97-N-inoculated tissues, the proportion of WO97-derived reads decreased from 2 to 4 wpi. Since the proportion of fungal-derived reads can be considered as a reference indicator of fungal activity and colonization status, this result suggests that the transcriptional activities of WO97-R and WO97-N were similar in orchid tissues at 2 wpi, but they differed at 4 wpi. Differences in the levels of fungal transcripts (the EF1α gene) among OM fungi-inoculated tissues were also assessed by reverse transcription quantitative polymerase chain reaction (RT-qPCR), which showed similar results to the proportion of fungal reads obtained by RNA-seq (**Supplementary Figure S2B**).

*To obtain gene expression data of Cypripedium* from the RNA-seq reads, read mapping to the reference and count estimations were conducted. We first used a contig set derived from *C. macranthos* var. *rebunense* transcriptomes for a reference, which has been constructed in a previous study (Assembly 4, Kambara et al., 2023); however, considering the mapping rate < 90%, we decided to create a new contig set from the short reads obtained in this study. The contig set constructed by integrating the short reads obtained from the five treatments is shown in **Table 1**. The number of contigs was 284,029 without removing WO97-derived short reads, while the number was 260,460 excluding the fungal sequences. The mapping rates remained at approximately 98% for both datasets. De-redundancy performed using CD-HIT-EST reduced approximately 100,000 contigs in both datasets, with improvements in complete single-copy BUSCO scores 32.0%–65.7% (including fungal sequences) or 33.8%–67.3% (excluding fungal sequences). The EvidentialGene program, used to reduce redundancy, further reduced the number of contigs and slightly decreased the mapping rate; it improved the BUSCO score by more than 87% (**Table 1**). Among the 88,236 contigs constructed from the short reads (excluding fungal reads), 3,388 contigs (3.4%) exhibited high sequence similarity to the CDS sequences of the WO97 draft genome and, hence, were excluded as fungal genes; a final set of 84,848 orchid-derived contigs were obtained for downstream analyses. The total mapping rate of the short reads (excluding fungal sequences) to 84,848 contigs was 87.4%, and the reduction in mapping rates was minimal (0.3%), confirming the reliability of the refined contig set. Among the 84,848 contigs, 23,694 contigs (27.92%) were annotated by the RefSeq plant database, 16,549 contigs (19.50%) were assigned GO terms, and 4,437 contigs (5.23%) were assigned KEGG pathway terms.

**Table 1:** Evaluation of constructed contigs before and after removal of fungi-derived reads.

Contig set (method)	Assembly contigs before fungal read removal				Assembly contigs after removing fungal reads			
	Contigs number	Mapping rate %	Busco (complete %)	Busco (complete–single copy %)	Contigs number	Mapping rate %	Busco (complete %)	Busco (complete–single copy %)
Trinity	284,029	98.1	94.0	32.0	260,460	98.2	93.2	33.8
Trinity + CD-HIT-EST	170,692	91.8	93.5	65.7	163,960	97.0	92.0	67.3
Trinity + evigene	97,454	87.4	92.7	85.5	88,236	87.7	92.3	87.0

Dataset **(A)** represents the assembly contigs before fungal read removal, whereas dataset **(B)** corresponds to the assembly contigs after removing OM fungal reads.

**Figure 3:**
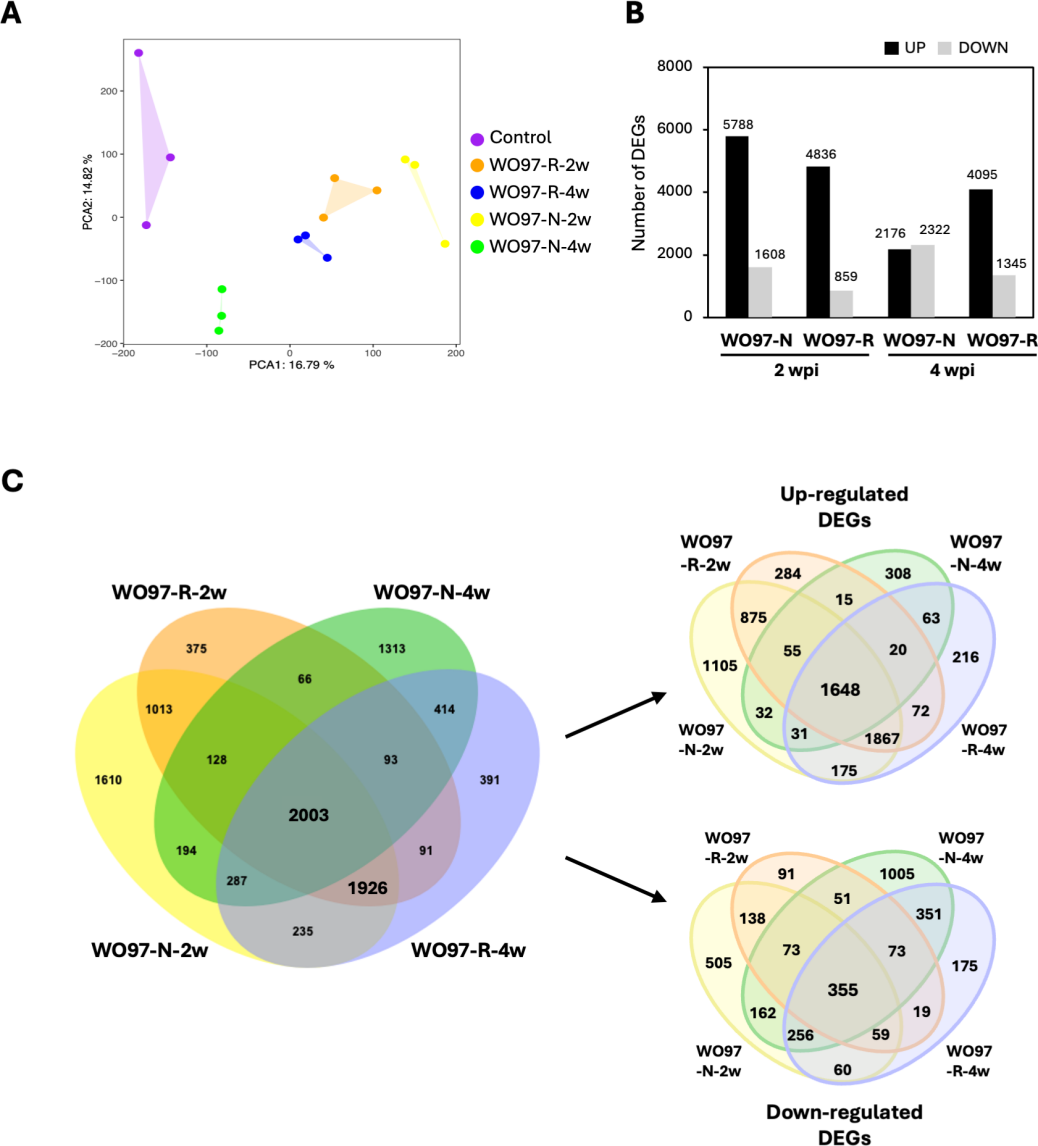
Expression profile and DEG analysis using fungus-inoculated *Cypripedium* root tissue. **(A)** Principal component analysis (PCA) of RNA-seq data using count data after the removal of the WO97-derived sequences. **(B)** The number of differentially expressed genes (DEGs) in each treatment group. The panel shows DEG counts after the removal of the WO97-derived sequences. **(C)** Venn diagram showing specific and shared DEGs across four comparative analyses. DEGs detected when using data after the removal of the WO97-derived sequences are used in this diagram.

The similarity in gene expression patterns among the samples was examined using count data via principal component analysis (PCA). As shown in **Figure 3A**, the five treatments (Control, WO97-R-2w, WO97-R-4w, WO97-N-2w, and WO97-N-4w) were distributed together with three replicate samples; the first principal component (PC1) explained 16.8% of the variance, and the second principal component (PC2) explained 14.8% of the variance. In the WO97-R-inoculated samples, the sample plots corresponding to 2 and 4 wpi were closely distributed, whereas they were far apart in the WO97-N-inoculated samples.

The number of DEGs derived by comparing gene expression levels between WO97-inoculated and uninoculated controls is shown in **Figure 3B**. In the WO97-N-2w, WO97-R-2w, and WO97-R-4w groups, the number of upregulated genes was greater than that of the downregulated genes. In contrast, in the WO97-N-4w group, the numbers of upregulated and downregulated genes were almost the same (**Figure 3B**). The finding that WO97-N-4w showed a different trend from the other three treatments was consistent with the PCA results, which is potentially related to the low abundance of fungus-derived transcripts in the WO97-N-4w group. The DEG analysis revealed that 10,139 genes expressed differently in the WO97-inoculated tissues compared with the control (before inoculation). The specificity and commonality of DEGs detected in the four comparisons are shown in **Figure 3C**. For further functional analyses, 2,003 and 1,926 DEGs were selected; the 2,003 DEGs shared in the four groups were predicted to be involved in the plant response to colonization by an OM fungus. The 1,926 DEGs shared only in the WO97-N-2w, WO97-R-2w, and WO97-R-4w groups were considered to be involved in adaptive mechanisms activated during continuous mycorrhizal interactions because their expression did not significantly change in the WO97-N-4w groups with reduced fungal activity. Among these DEG sets, 51.8% of the 2,003 DEGs and 30.1% of the 1,926 DEGs were annotated using the RefSeq plant database.

### Functional insights into the detected DEGs

**Figure 4:**
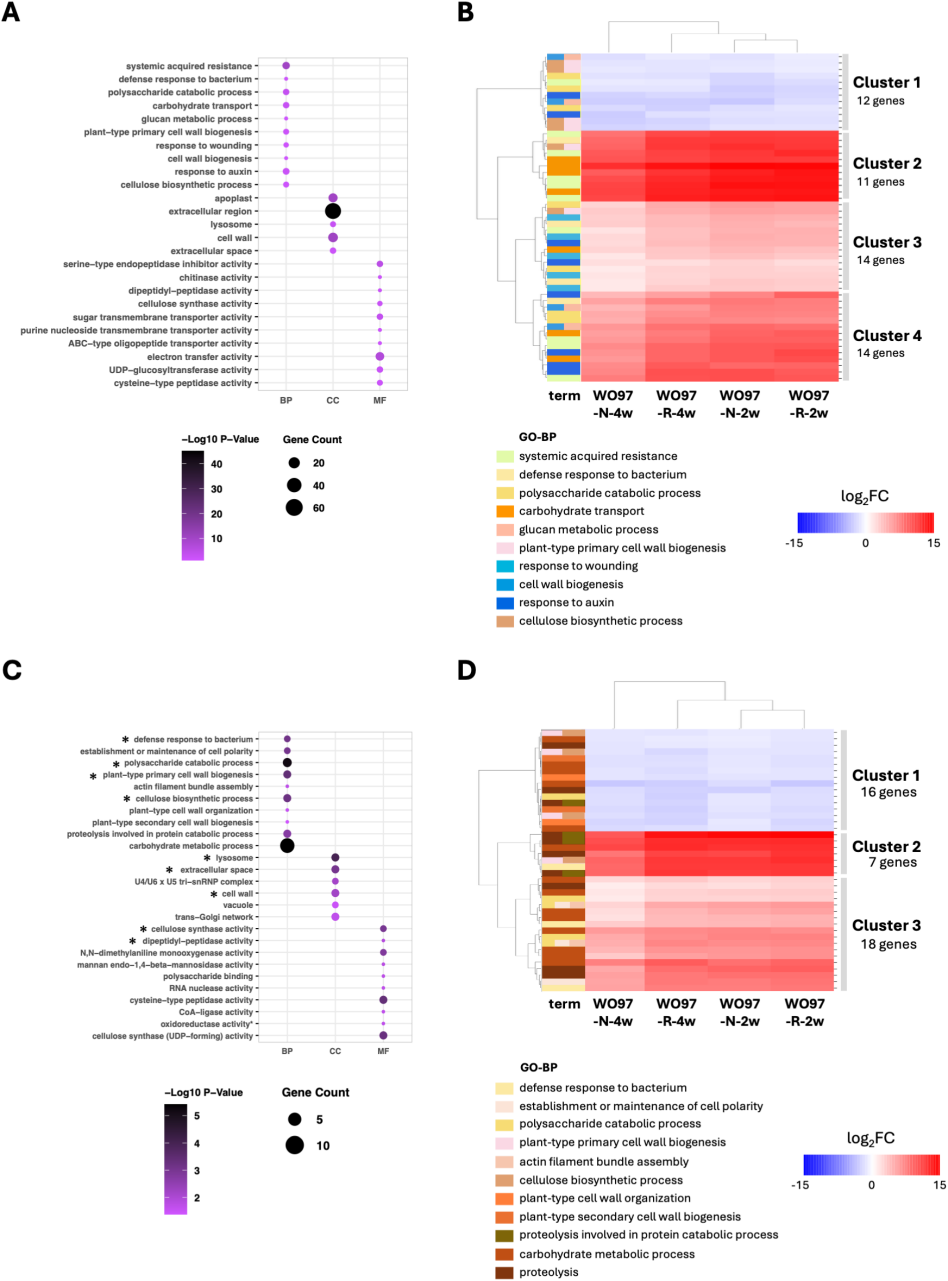
GO enrichment analysis of the 2,003 DEGs. **(A)** GO terms significantly enriched under “biological process” (BP), “cellular component” (CC), and “molecular function” (MF) with the 2,003 shared DEGs across all four conditions (WO97-R-2w, WO97-N-2w, WO97-R-4w, and WO97-N-4w). The *y*-axis lists the GO terms (at most the top 10) ranked based on enrichment value. **(B)** The heatmap of gene expression patterns reflecting the enriched 10 GO biological process (BP)-related terms associated with 2,003 shared DEGs. **(C)** GO enrichment results for the 239 genes identified through PPI processing of the 2,003 DEGs. An asterisk (*) indicates GO terms that remained enriched before and after PPI processing. **(D)** The heatmap shows the expression patterns of genes associated with the enriched 11 GO BP terms associated with 239 genes. Analyses were conducted using the GO.db package in R. The heatmap was generated using Seaborn and Matplotlib in Python. Annotation and relative expression levels of each gene in **(B, D)** are shown in **Supplementary Tables S1** and **S2**, respectively.

GO and KEGG enrichment analyses conducted for studying the functional roles of DEGs across the four comparisons revealed that genes annotated with enriched GO terms comprised 44.0% of the 2003 up- and downregulated DEGs, whereas only 10.0% of DEGs were annotated with enriched KEGG pathway terms. The top five enriched and significant GO BP terms with the lowest adjusted *p*-values (*P*_adj_) were “systemic acquired resistance”, “defense response to bacterium”, “polysaccharide catabolic process”, “carbohydrate transport”, and “glucan metabolic process” (**Figure 4A**). In the KEGG pathway enrichment, “cutin, suberine and wax biosynthesis”, “platinum drug resistance”, “ABC transporters”, “synaptic vesicle cycle”, and “fructose and mannose metabolism” were detected (**Supplementary Figure S3A**), but no significance was detected due to low *P*_adj_ values. Among the 10,139 DEGs, 51 genes were annotated with the significantly enriched 10 GO-BP terms. Heatmap clustering of the 51 genes showed that the DEGs designated in Clusters 2 and 4 were strongly upregulated in OM fungi-colonized tissues when compared to noncolonized controls (**Figure 4B**); these clusters included *SWEET* sugar transporters, cell wall modification-related proteins, and enzymes such as expansin-like protein (EG45-like domain-containing protein), glucan 1,3-beta-glucosidase, cellulose synthase catalytic subunit, and xyloglucan endotransglucosylase/hydrolase (XTH) (**Supplementary Table S1**). The pathogenesis-related protein (PR4) and L-type lectin receptor-like kinase (LecRLK) were also included in the upregulated gene clusters. Additionally, the 12 DEGs designated in Cluster 1 were consistently downregulated across all fungi-colonized tissues; the downregulated genes included cellulose synthase catalytic subunit and *XTH* genes (**Supplementary Table S1**), suggesting a fine-tuned regulation of the physical properties of the cell wall in response to OM fungal colonization. Regarding genes related to plant hormones, auxin-responsive *SAUR* genes were detected in Clusters 3 and 4, which are clusters of upregulated genes.

We used the PPI network constructed using STRING to identify key genes crucial for cellular signaling events, among the detected 2,003 DEGs. The PPI network resulted in 830 nodes and 2,160 edges with significant enrichment (*p* < 1.0e-16) (**Supplementary Figure S4**). Further analysis via Cytoscape using two centrality parameters (Degree and Betweenness) identified key genes ranking in the top 30% of the two centrality values, and the obtained 239 genes were used for post-PPI GO enrichment analysis. The significantly enriched GO terms (BP, CC, and MF) associated with the 239 genes are shown in **Figure 4C**; in the BP terms, “defense response to bacterium”, “polysaccharide catabolic process”, “plant-type cell wall biogenesis”, and “cellulose biosynthetic process” were again detected as enriched terms. Furthermore, it included newly detected terms, such as “establishment or maintenance of cell polarity” and “actin filament bundle assembly”, and cell wall metabolism-related terms, such as “plant-type primary cell wall organization” and “plant-type secondary cell wall biogenesis”. The relative expression levels of the DEGs annotated with the enriched 11 GO-BP terms are shown in **Figure 4D** and **Supplementary Table S2**. Genes related to the polysaccharide catabolic process or carbohydrate metabolic processes were included in both the upregulated cluster (Cluster 3, **Figure 4D** and **Supplementary Table S2**) and the downregulated cluster (Cluster 1, **Figure 4D** and **Supplementary Table S2**), indicating their involvement in the adjustment of cellular architecture to support the establishment of the mycorrhizal structure. The defense response-related genes *PR-4* (Cluster 2) and *LecRLK* (Cluster 3) were consistently retained as upregulated genes before and after PPI processing, highlighting their potential role in regulating defense-related responses in mycorrhizal interactions. Besides BP terms, cellular component (CC) terms such as “lysosome”, “extracellular space”, and “cell wall” were retained after PPI filtering, along with MF-related terms including “cellulose synthase activity” and “dipeptidyl-peptidase activity.”

**Figure 5:**
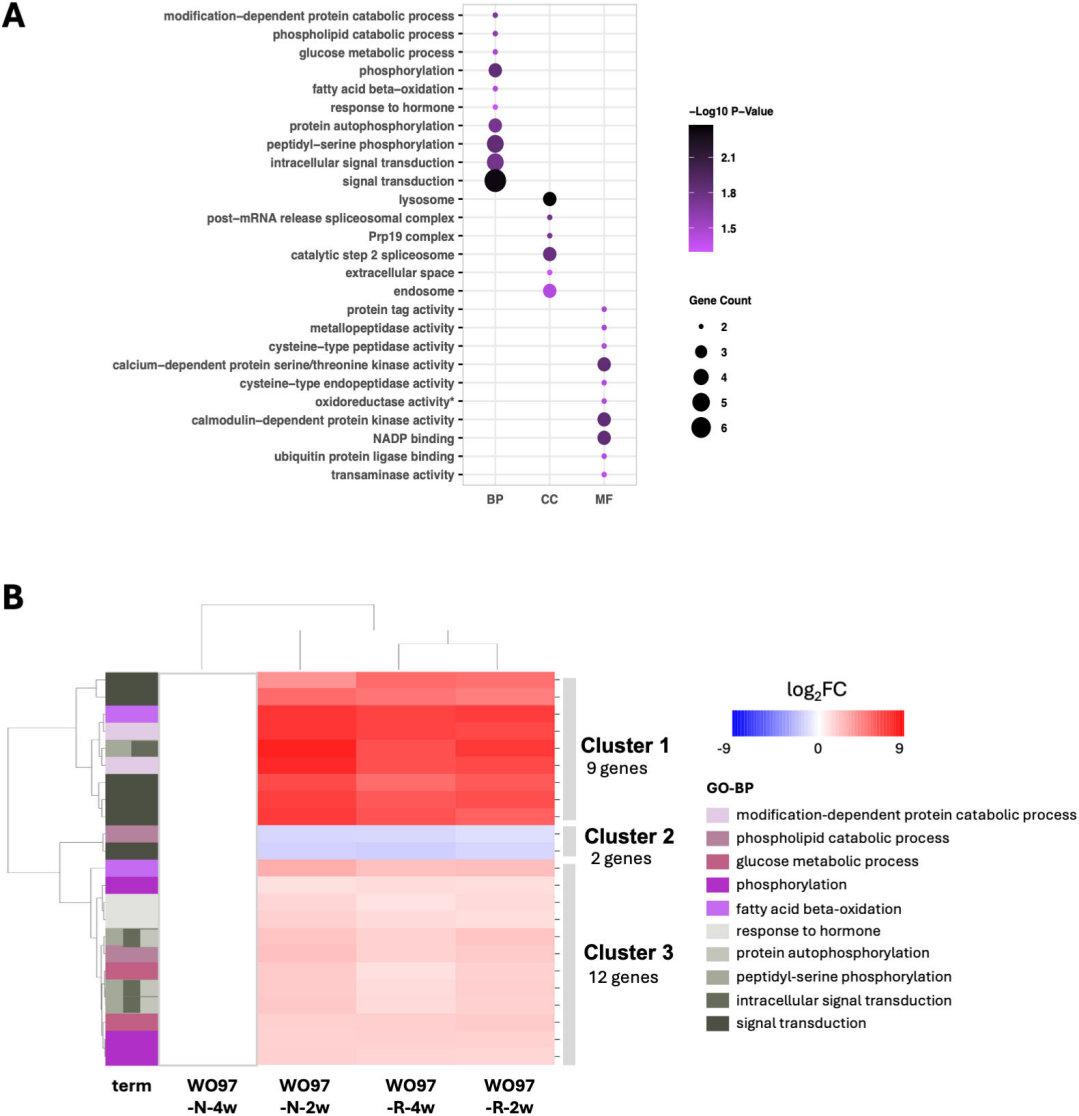
Heatmap of gene expression patterns reflecting enriched GO biological process-related terms associated with 1,926 shared DEGs. **(A)** GO enrichment results for the 143 genes identified through PPI processing of the 1,926 DEGs. The *y*-axis lists the GO terms (at most the top 10) ranked based on enrichment value. **(B)** The heatmap shows the expression patterns of genes associated with the enriched 10 GO terms in the set of 143 genes obtained after PPI processing for the 1,926 DEGs. The heatmap was generated using Seaborn and Matplotlib in Python. Annotation and relative expression levels of each gene in **(B)** are shown in **Supplementary Table S2**.

Next, we conducted GO and KEGG enrichment analyses using the 1,926 DEGs shared among the three conditions, except for the WO97-N-4w group. Genes annotated with enriched GO terms comprised 32.2% of the 1,926 DEGs, whereas only 5.3% of them were annotated with enriched KEGG pathway terms. GO-BP terms “leaf development”, “histidine biosynthetic process”, “trehalose metabolism in response to stress”, “endoplasmic reticulum to cytosol auxin transport”, and “NLS-bearing protein import into nucleus” were detected from the 1,926 DEGs (**Supplementary Figure S5**), but no significance was obtained due to low *P*_adj_ values. In the KEGG pathway enrichment, four of the top five pathway terms with the lowest *P*_adj_ values detected in 2,003 DEGs (“Platinum drug resistance”, “ABC transporters”, “Synaptic vesicle cycle”, and “Fructose and mannose metabolism”) also appeared in 1,926 DEGs (**Supplementary Figure S3B**), but significance of the enrichment of pathway terms was not detected in the 1,926 DEGs, as in the case of the 2,003 DEGs. Using the 1,926 DEGs, a PPI network with 487 nodes and 1,386 edges was constructed (PPI enrichment, *p* = 5.22e-05) (**Supplementary Figure S6**); 143 genes (top 30%) were included in the PPI network with significant centrality values and were used for GO analysis. Through the PPI filtering process, the GO terms were significantly enriched in the 143 genes (**Figure 5A**). Notably, “phosphorylation”- and “signal transduction”-related terms emerged as enriched GO-BP terms. Heatmap clustering and annotation of the 23 genes annotated with enriched 10 GO-BP terms are depicted in **Figure 5B** and **Supplementary Table S2**. Several kinase genes, including serine/threonine-protein kinase (STY46), mitogen-activated protein kinase kinase kinase 18 (MAPKKK18), histidine kinase 1 (HK1), and calcium-dependent protein kinases were abundantly expressed in WO97-N-2w, WO97-R-2w, and WO97-R-4w, highlighting phosphorylation events in intracellular signaling cascades that coordinate host responses during continuous mycorrhizal interactions (**Figure 5B**; **Supplementary Table S2**). Although its expression level was lower than that of the kinase genes in Cluster 1, the auxin response factor *ARF9* genes were included in the upregulated genes in Cluster 3 (**Supplementary Table S2**). In CC terms, “lysosome”, “post-mRNA release spliceosomal complex”, “Prp19 complex”, and “extracellular space” were detected in enrichment after PPI filtering. In MF terms, terms related to various enzyme activities were enriched in addition to the kinases.

### Variable expression of transporter genes in mycorrhizal tissue

To investigate the potential roles of transporters in facilitating nutrient exchange under varying levels of mycorrhizal status and fungal activity, we examined the differential expression of transporter genes included in the 2,003 and 1,926 DEGs among the four conditions. A total of 58 and 31 transporter genes were identified among the sets of 2,003 and 1,926 DEGs, respectively, most of which were upregulated in both sets, highlighting their active involvement in nutrient exchange and cellular adaptation mechanisms during orchid and OM fungi interactions (**Supplementary Figure S7**). Among the transporter genes, a notable abundance of ABC transporters across both DEG sets suggested their essential role in transferring a variety of substrates such as lipids and phytohormones in response to OM fungus colonization. The sugar transporters *SWEET4* and *SWEET14* were included in the set of 2,003 DEGs, but only *SWEET1* was included in the set of 1,926 DEGs; hence, the functions of these SWEETs may vary depending on mycorrhizal status. Transporter genes for organic nitrogen-containing compounds such as amino acids, peptides, and nucleic acids were upregulated by OM fungal colonization, and several genes were expressed only in 1,926 DEGs. Searching for transporters of inorganic nitrogen compounds, one ammonium transporter and several nitrate transporter genes were found to be upregulated among the 2,003 DEGs. Notably, the expression levels of the transporter genes in the WO97-N-4w group were lower than those in the other three groups, with expression patterns corresponding to changes in fungal activity and nutrient availability across the different colonization stages.

### Temporal and hyphal culture-specific gene expression changes

**Figure 6:**
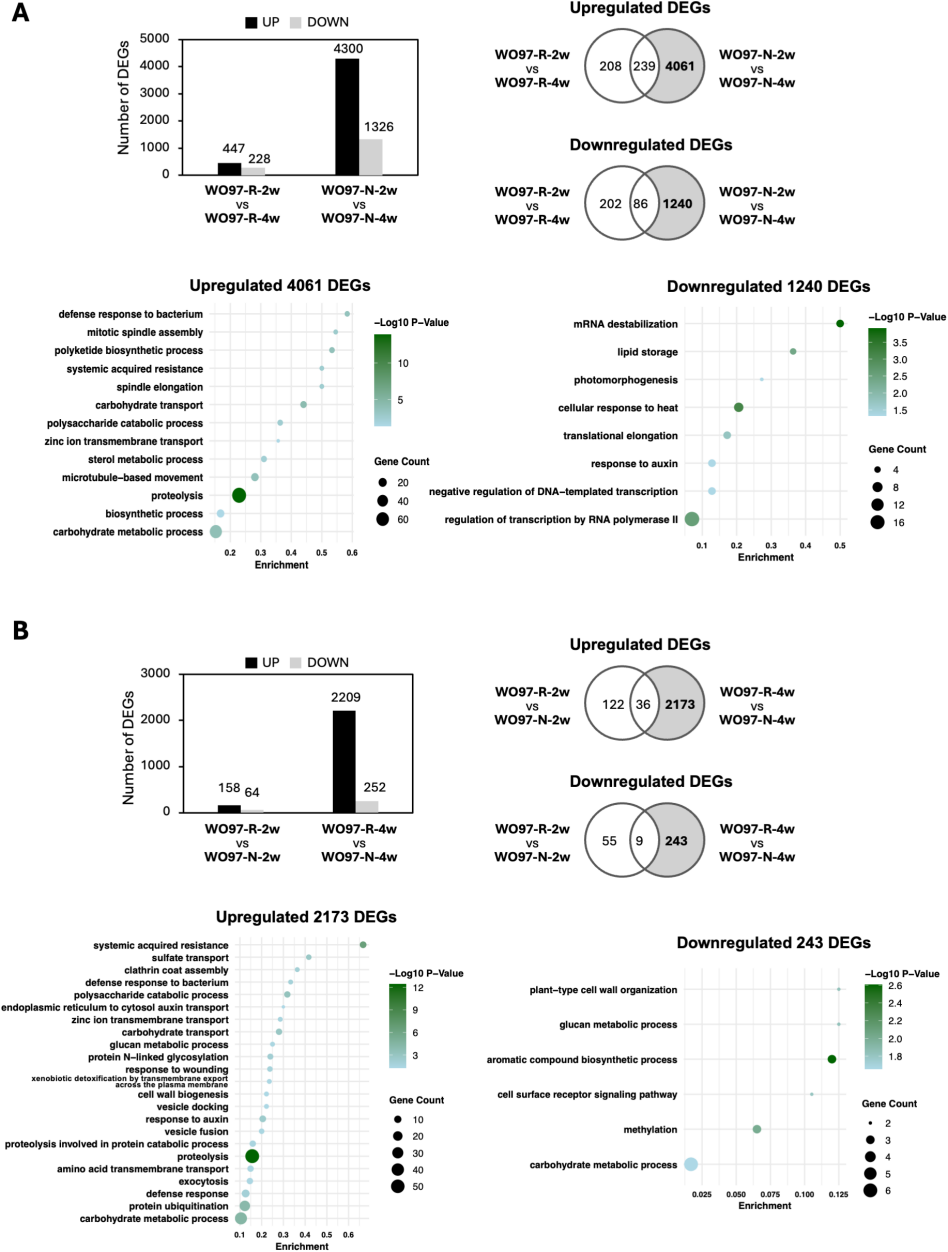
Temporal and hyphal culture-specific variation in DEGs and functional enrichment of the detected DEGs. The number of DEGs between two time points (2 wpi vs 4 wpi) **(A)** and between the two fungal cultures (WO97-R vs. WO97-N) **(B)**. Venn diagrams show the overlap of DEGs in both comparisons. GO enrichment analysis was performed on the DEGs highlighted in the gray-shaded section of the Venn diagram. The significantly enriched biological process (BP) terms with *P*_adj_ values (< 0.05) are presented. Each annotation of gene with an enriched GO term is shown in **Supplementary Table S3**.

To further elucidate the changes in gene expression during mycorrhizal interactions, we analyzed the DEGs across two time points (2 wpi vs. 4 wpi) (**Figure 6A**) and after inoculation with two fungal cultures (WO97-R vs. WO97-N) (**Figure 6B**). The DEG analysis revealed distinct gene expression patterns associated with temporal progression of fungal colonization and the different fungal subcultures. The temporal comparison (2 wpi vs. 4 wpi in WO97-R or WO97-N) revealed that the number of detected DEGs was significantly higher in the WO97-N-colonized tissues compared to WO97-R-colonized tissues; in the WO97-N-colonized tissues, 4,300 DEGs were upregulated and 1,326 DEGs were downregulated genes at 2 wpi compared with 4 wpi, while 447 DEGs were upregulated and 288 DEGs were downregulated at 2 wpi compared when 4 wpi in the WO97-R-colonized tissues (**Figure 6A**). The DEGs upregulated specifically in WO97-N-colonized tissues (4,061 DEGs) at 2 wpi were enriched significantly in biological process terms “defense response to bacterium”, “mitotic spindle assembly”, “polyketide biosynthetic process”, and “systemic acquired resistance” (**Figure 6A**). A total of 33 genes were associated with the top five GO terms with the lowest *P*_adj_ values, including *LecRLK*, *PR4*, *kinesin-like protein*, and *EG45-like domain containing protein* genes (**Supplementary Table S3**). The comparative analysis of WO97-R vs. WO97-N at the same time point after the second inoculation showed that very few DEGs (222 DEGs) were detected at 2 wpi, whereas 2,461 DEGs were detected at 4 wpi; most DEGs were significantly less expressed in WO97-N-colonized tissues than in WO97-R-colonized tissues at 4 wpi (**Figure 6B**). The 2,173 genes specifically upregulated in WO97-R-colonized tissues at 4 wpi were associated with defense-related GO terms; *LecRLK*, *PR4*, and *EG45-like domain containing protein* genes, as well as *sulfate transporter* and *clathrin assembly protein* genes, were associated with the top five GO enriched terms with the lowest *P*_adj_ values (**Supplementary Table S3**). On the other hand, 1,240 DEGs whose expression levels were lower at 2 wpi than at 4 wpi in WO97-N-colonized tissues (i.e., upregulated genes at 4 wpi compared to 2 wpi in WO97-N-colonized tissues) were enriched in several GO terms including “mRNA destabilization”, “lipid storage”, “photomorphogenesis”, “cellular response to heat”, and “translational elongation”, which suggests a potential reduction in metabolic demand and stress-related responses, coinciding with a decrease in fungal activity and the disappearance of peloton formation.

## Discussion

Transcriptomic analyses of plant–microbe interactions face unique challenges owing to the mixing of transcripts derived from different organisms associated with the interaction. If the genome or gene-coding sequences are well determined, short reads of different origins can be distinguished and analyzed separately. In some previously reported transcriptome studies dealing with orchid–OM fungi interactions, fungi-derived reads were analyzed separately (Miura et al., 2018; Valadares et al., 2021), whereas separate analysis was not possible in others (Tsai et al., 2016; Schelkunov et al., 2018; Gao et al., 2022). In the present study, the genome sequence of *Cypripedium* sp. was not available; therefore, we generated a reference gene set by *de novo* assembly using short reads. In the mycorrhizal tissue materials, hyphal growth and peloton formation differed between the two WO97 cultures at 4 wpi (**Figure 1**; **Supplementary Figure S2**); therefore, we examined the proportion of fungal-derived short reads in the total reads obtained via RNA-seq and found different proportions of reads mapped to the WO97 draft genome (i.e., WO97-derived transcripts) across samples (**Figure 2**). Despite the low frequency (approximately 10% of the total reads), fungal reads influenced subsequent analysis; therefore, removing as many microbe-derived reads as possible can enhance the accuracy of plant gene expression analysis using colonized tissues; in fact, the PCA result and number of detected DEGs differed considerably between the analyses including WO97-derived reads and those excluding fungal reads, especially in the analysis for WO97-N-4w (data not shown). In this study, the proportion of fungal reads (approximately 8%–14%) derived using the orchid root tissue exhibiting peloton formation was comparable to the proportion of fungal reads detected in the germinated protocorm tissue of other orchid species (Miura et al., 2018). Transcriptome analysis of orchid roots growing under natural conditions revealed 5.1%–18.3% and 2.2%–4.7% fungi-derived reads in roots with and without mycorrhizae, respectively (Valadares et al., 2021); it is suggested that mycorrhizal interactions in orchid tissues can be associated with fungal transcripts varying from 2% to 18% of the total transcripts in the tissue. We employed a two-step strategy to remove fungal sequences from both short reads and constructed contig sets, which enabled a refined analysis of transcriptional changes in the host orchid *C. macranthos* var. *rebunens*e during mycorrhizal interactions. Although the assembly processing was conducted after excluding short reads that were mapped to the fungal genome, the constructed contig set unexpectedly contained sequences annotated as fungal genes. This may be because the genome sequence of the fungus used was not complete (draft genome) and/or short reads may not be able to accurately distinguish between fungal and orchid gene sequences.

Genomic resources for orchids are limited, with only a few species having their genomic information available (e.g., *D. catenatum*, *P. equestris*, *A. shenzhenica*, two *Platanthera* species, and *O.* sp*hegodes*), which encode approximately 21,841–29,431 genes; *O.* sp*hegodes* has 42,549 annotated genes (Cai et al., 2015; Zhang et al., 2016, 2017; Li et al., 2022; Russo et al., 2024). Orchidaceae is an exceptionally diverse plant family exhibiting substantial genomic variation among species. The number of genes estimated via transcriptomic studies is greater than that via genomic analysis; for instance, 59,143 non-redundant contigs in *Bletilla striata* and 30,591 transcripts (assigned as Streptophyta) in *Limodorum abortivum* were reported (Miura et al., 2018; Valadares et al., 2021). In this study, we obtained 84,848 contigs as unigene sets after removing WO97-derived sequences and redundancy removal. While the subfamily Cypripedioideae is known to possess the largest genome size among orchid subfamilies (Leitch et al., 2009), publicly available reference genome sequences for species within the genus *Cypripedium* are still limited. Considering that only 27.92% of the contig set obtained in this study were annotated as known plant genes, this gene set is still likely to contain unknown information. Genome sequencing requires high-quality, high-molecular-weight genomic DNA and appropriate extraction methods, which require more plant tissues and preliminary experiments. In the future, genome sequence analysis using long-read sequencing in *Cypripedium* should be considered for analyzing gene expression profiles with improved accuracy.

Among the significantly enriched GO terms associated with DEGs shared by all tested OM fungus-colonized conditions (WO97-R-2w, WO97-R-4w, WO97-N-2w, and WO97-N-4w), the genes with considerably high expression levels included *SWEET4*, *SWEET14*, *cellulose synthase*, *EG45-like domain containing protein (expansin)*, *xyloglucan endotransglucosylase/hydrolase (XTH)*, and *pathogenesis-related protein 4 (PR4)* genes (Cluster 2 in **Figure 4B** and **Supplementary Table S1**). The expression of genes involved in cell wall remodeling and sugar transport was significantly increased during colonization by an OM fungus, suggesting their involvement in changes in the physical properties of cell wall components and the supply of nutrients necessary for the establishment of mycorrhizal structures. In addition, we focused on genes related to PPIs, which play important roles in the regulation of gene expression and intracellular structure. Among the genes narrowed down by constructing a STRING-based PPI network are those encoding PR4, acidic chitinase, cellulose synthase, and proteases (SAG39-like and cucumisin) and those that showed particularly high expression levels (Cluster 2 in **Figure 4D** and **Supplementary Table S2**). The PR4 protein family contains chitin-binding motifs and is considered to contribute to defense responses against plant-infecting fungi through chitinase and RNase activities (Franco et al., 2019; Dabravolski and Frankel, 2021). In addition to the chitinase gene, genes encoding hydrolase of β-1,3-glucan, the major polysaccharide in the fungal cell wall, and plant cell wall remodeling enzyme genes were also detected as upregulated genes in the PPI network. L-type lectin receptor-like kinases (LecRLKs) were also detected as upregulated genes in the PPI network. LecRLKs, a plant-specific receptor-like kinase (RLK) subfamily, have been recently found to play crucial roles in plant development and responses to abiotic and biotic stresses (Navarro-Gochicoa et al., 2003; Sun et al., 2020). The G-type LecRLK interacts with Nod factors during rhizobial symbiosis, and the L-type LecRLK in *Arabidopsis* positively regulates PAMP-triggered immunity (Sun et al., 2020). Although the function of LecRLK in OM interactions remains unknown, it is likely that this membrane-localized receptor kinase senses OM fungal colonization and regulates gene expression correlated with cell wall remodeling, nutrient transport required for peloton formation, and mycorrhizal interactions. The shared DEGs in the four treatments were discussed above; however, considering that fungus-derived transcripts were reduced in WO97-N-4w, the expression changes of these genes may be a general response to fungal infection in orchid tissues, regardless of mycorrhizal interaction. In the GO enrichment analysis using the 1,926 DEGs, signal transduction-related genes (*STY46*, *MAPKKK18*, and *histidine kinase 1*) were identified to be upregulated genes (Cluster 1 in **Figure 5B** and **Supplementary Table S2**) by focusing on genes in PPI networks. STY46 suppresses plant defense against the pathogenic fungus *Botrytis cinerea* infection, which suggests its negative role in chitin-induced immunity (Chen et al., 2024). MAPKKK18 triggers a phosphorylation cascade that initiates chitin-related defense responses in *Oryza sativa* (Wang et al., 2017). Although the specific function of these kinases in orchids remains unexplored, we hypothesize that mycorrhizal colonization recognized by LecRLKs is signaled by these kinases (STY46, MAPKKK18, and histidine kinase 1) and the defense responses are fine-tuned to maintain an appropriate mycorrhizal interaction. The results of the GO enrichment analysis suggest possible roles for the associated genes; however, owing to the low annotation rate and limited number of significantly enriched terms, further validation is required to confirm these hypotheses.

In the DEGs analyses between WO97-N-2w and WO97-N-4w or WO97-R-4w and WO97-N-4w (**Figure 6**), defense- and signaling-related genes (e.g., *LecRLK*, *PR4*, and *EG45-like domain containing protein*) and genes related to carbohydrate transport and metabolic process exhibited notably reduced expression at WO97-N-4 wpi. These results suggest that defense-related responses and gene expression related to cell structure modification were even activated in WO97-N-colonized tissues at 4 wpi compared to noncolonized controls, but their expression levels were not as high as in mycorrhizal tissues. The number of DEGs between WO97-R- and WO97-N-colonized tissues at 2 wpi was low, and there was little difference in gene expression between these issues. It seems that the fungal abundance is greater in WO97-N-infected tissues than in WO97-R-infected tissues at 2 wpi (**Figure 2**; **Supplementary Figure S2B**), and further analyses are needed to clarify whether this difference is related to the significant differences in host gene expression after 2 weeks.

WO97 is an unidentified species belonging to the genus *Tulasnella* (Shimura et al., 2009); unlike AM mycorrhizal fungi, *Tulasnella* spp. lack genes involved in nitrate uptake (Fochi et al., 2017). It has been suggested that *Tulasnella calospora*, an OM fungus associated with *Serapias vomeracea*, uptakes NH_4_^+^ from the mycorrhizal interface via the fungal ammonium transporter and that *S. vomeracea* exchanges organic nitrogen compounds such as amino acids and peptides with its OM fungus (Fochi et al., 2017). Our transcriptomic study revealed that the expression of many genes involved in nutrient transport was increased in response to OM fungal colonization (**Supplementary Figure S7**); the upregulated genes included nitrate transporters and transporters of many organic nitrogen-containing compounds (amino acids, peptides, and nucleic acids). It is suggested that *Cypripedium* also exchanges organic nitrogen compounds with the fungus; however, further research is needed to clarify the details of this nutrient exchange.

In this study, we examined gene expression related to mycorrhizal interaction in *C. macranthos* var. *rebunense* using two subcultures (WO97-R and WO97-N), with each inducing a different gene expression in the colonized orchid seedlings. In WO97-R-inoculated tissues, genes upregulated at 2 wpi were also upregulated at 4 wpi, whereas in WO97-N-inoculated tissues, expression of many genes upregulated at 2 wpi was not maintained at 4 wpi. WO97-R and WO97-N were derived from the same WO97 strain and shared identical ITS1 and ITS2 sequences. The properties of vegetatively propagated fungi can change during subculture, and epigenetic changes may be involved, but detailed mechanisms responsible for the different mycorrhizal interaction properties of strains multiplied by subculture remain largely unknown. In the orchid’s research on symbiotic germination and mycorrhizal interaction with the genus *Tulasnella*, *T. calospora* is commonly used because this species is a mycorrhizal partner for many terrestrial orchids and generally exhibits relatively stable symbiotic behavior. The rDNA-ITS sequence (Shimura et al., 2009) and the genome sequence (our unpublished data) revealed that WO97 and *T. calospora* are phylogenetically distant within the genus *Tulasnella*; therefore, it is possible that these *Tulasnella* species exhibit different physiological properties when interacting with host orchids. It has been reported that the Tulasnellaceae is a genetically diverse group and that even closely related *Tulasnella* strains vary in morphology and compatibility with host orchids (Curz et al., 2011; Taylor and McCormick, 2008; Fuji et al., 2020). Among the fungi of the Tulasnellaceae family, *T. calospora* is a generalist as an OM fungus that is well-adapted to many terrestrial orchid species, while *Tulasnella* groups adapted to *Cypripedium* may exhibit different relationships with variable host compatibility. Further research is also needed to understand the differences in the physiological characteristics involved in the diversity of mycorrhizal interactions among *Tulasnella* species.

## Data Availability

Data obtained by RNA-seq are available the NCBI SRA database with accession number PRJNA1231755.
