# Supplementary material for: Transcriptomic and protein–protein interaction network analyses of the molecular mechanisms underlying the mycorrhizal interaction in Cypripedium macranthos var. rebunense
Source: Front Plant Sci. 2025 Jul 10;16:1597154. doi: 10.3389/fpls.2025.1597154 (PMC12287105; doi:10.3389/fpls.2025.1597154)
Supplement: Supplementary file 1 [file DataSheet1.pdf]

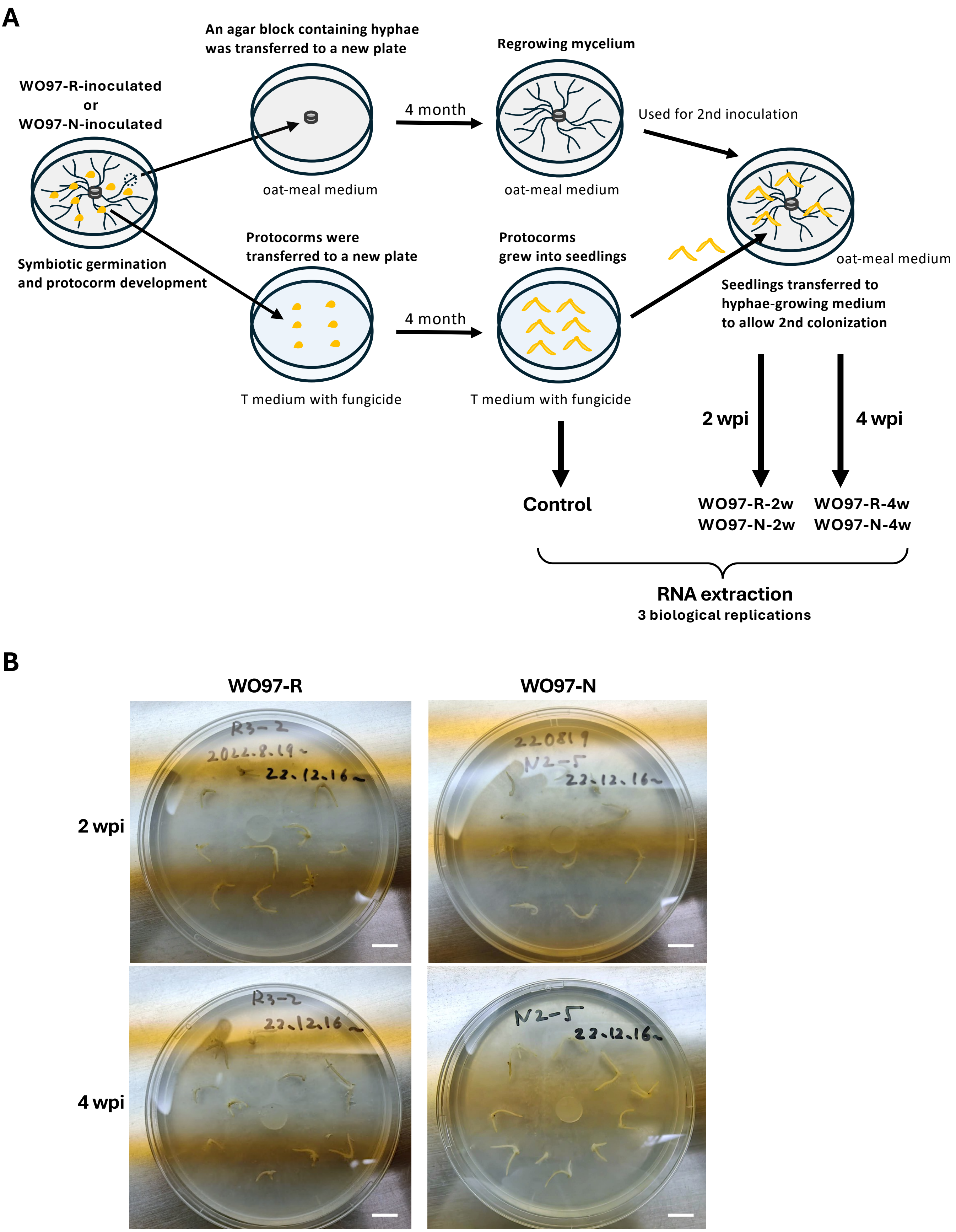

Suppelementary Figure S1 The experimental setup for preparing materials for RNA-seq. (A) Flow of sample preparation for RNA-seq. Protocorms transfer and the inoculum preparation for the second inoculation were performed in the same way for both WO97-R and WO97-N. (B) Appearance of seedlings before sampling. No difference in growth was observed between seedlings inoculated with WO97-R or WO97-N during incubation with the fungus. Bars, 1 cm

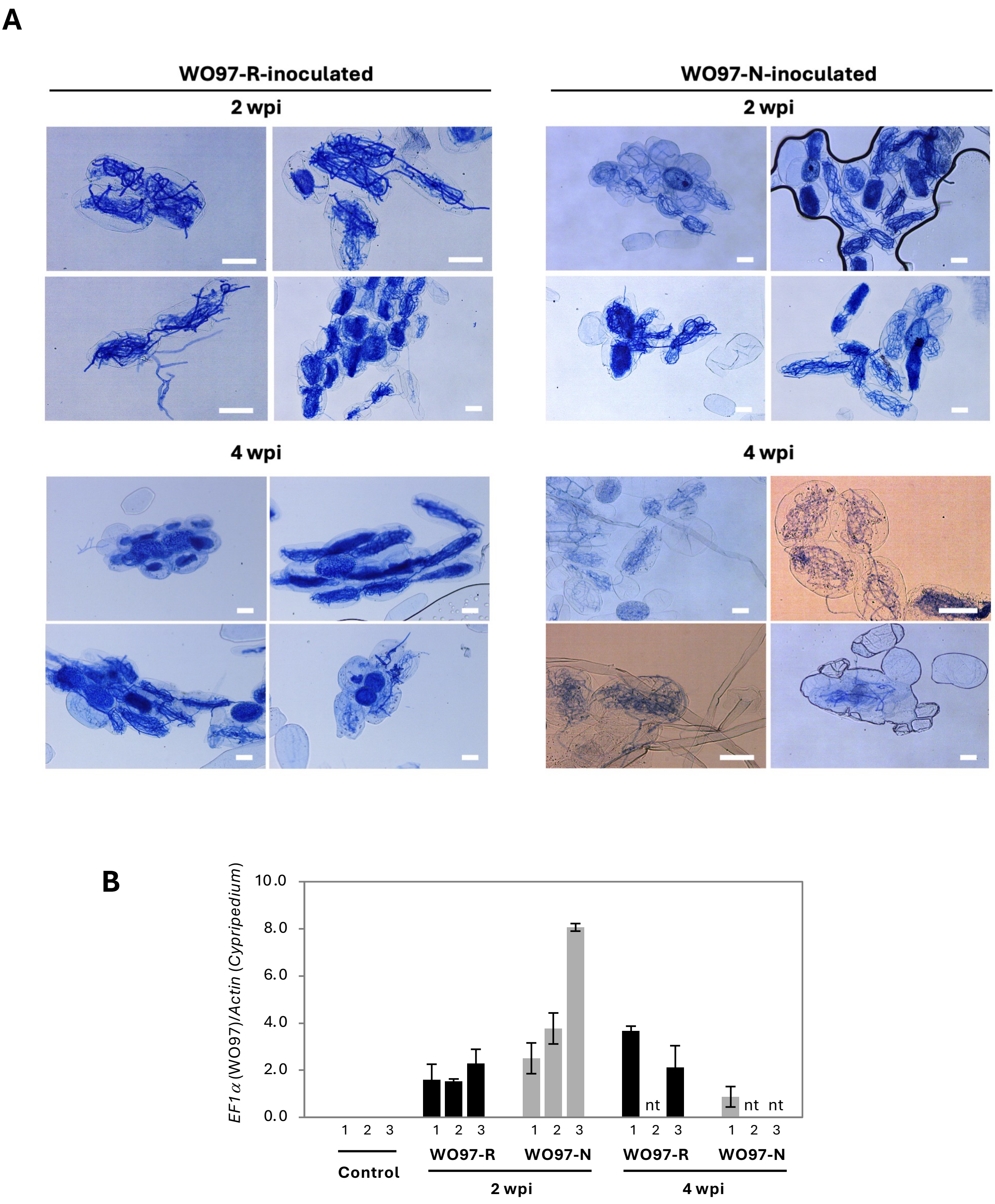

Suppelementary Figure S2 Fungal colonization in the root tissues of *Cypripedium macranthos* var. *rebunense* seedlings inoculated with a mycorrhizal fungus (WO97-R or WO97-N). (A) Hyphal coil formation (peloton formation) in the tissues. Root tissues were observed 2- and 4- weeks post inoculation (wpi). Bars, 50 μm. (B) Fungal gene expression levels in the inoculated tissues. Expression levels of the *EF1α* gene of WO97 relative to the *actin* gene expression levels in *C. macranthos* var. *rebunense* evaluated by RT-qPCR. The *EF1α* gene expression was not detected in the Control (seedling tissues before inoculation). nt, samples not evaluated due to lack of RNAs. Although WO97-N-2w tends to have higher fungal *EF1a* expression than WO97-R-2w,, but no statistically significant difference was detected between the average values ($p = 0.15$).

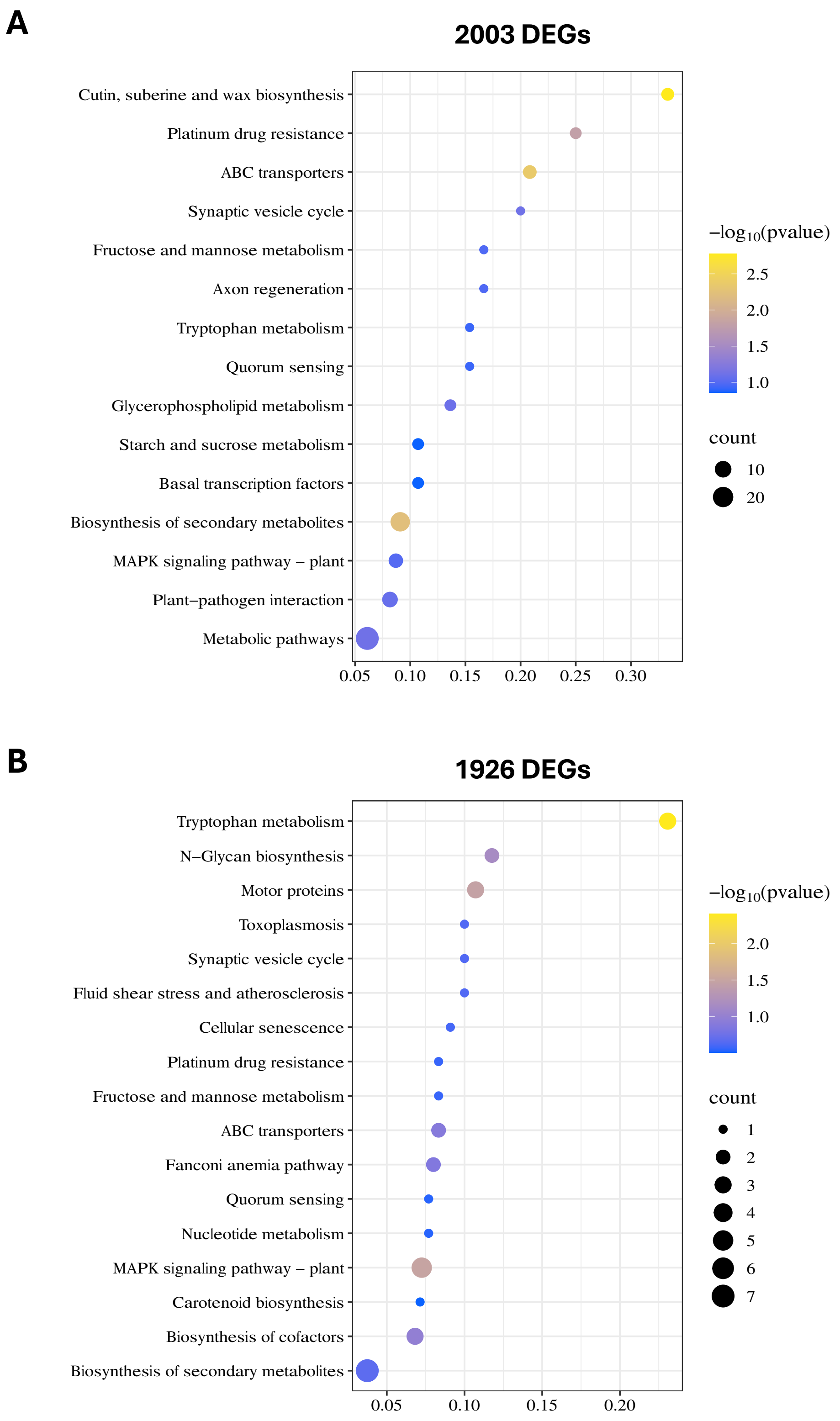

Suppelementary Figure S3. Detection of KEGG pathway annotations among 2,003 DEGs set (A) and 1,926 DEGs set (B).

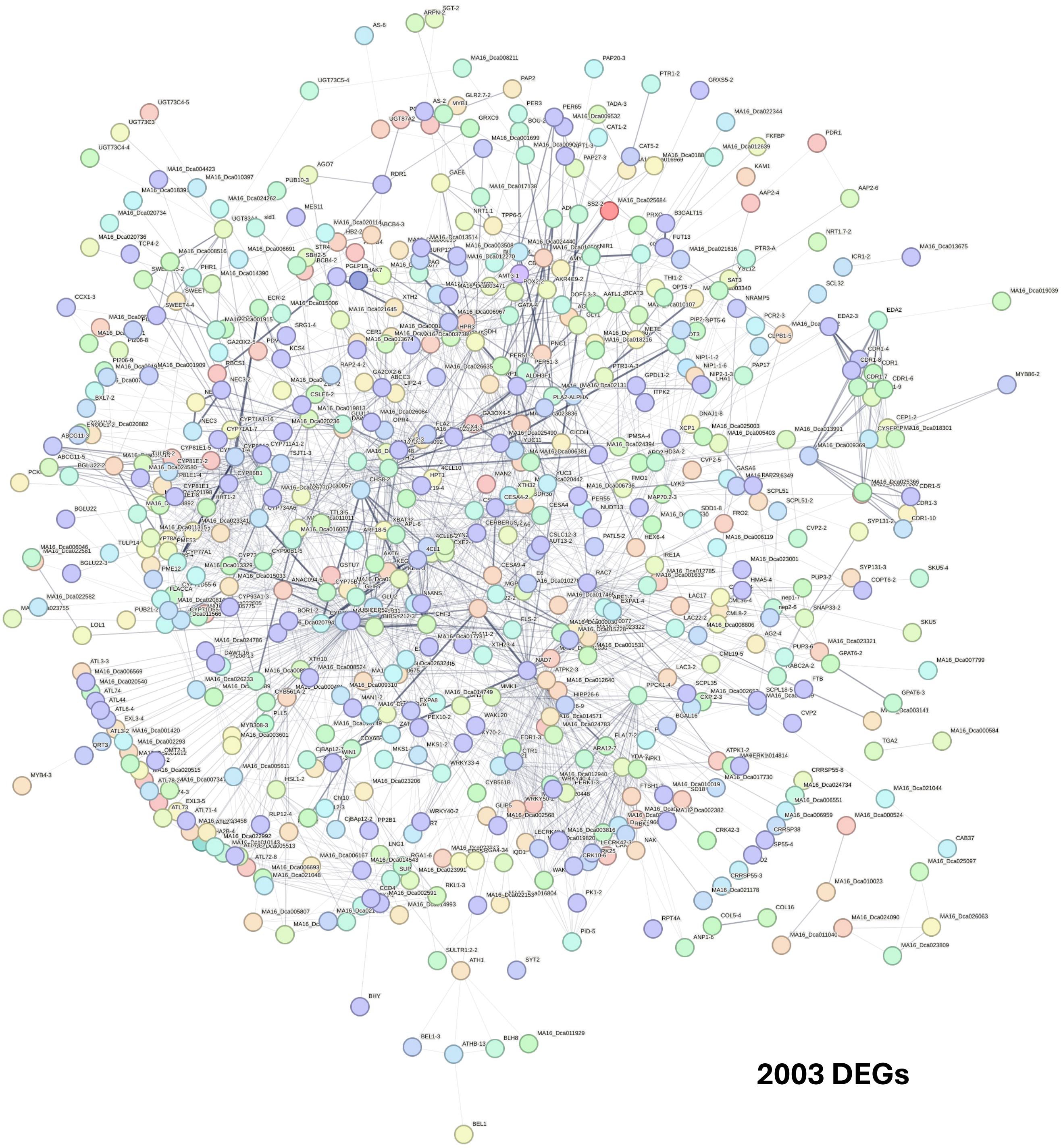

Suppelementary Figure S4 Protein-protein interaction (PPI) network constructed using 2,003 shared DEGs.

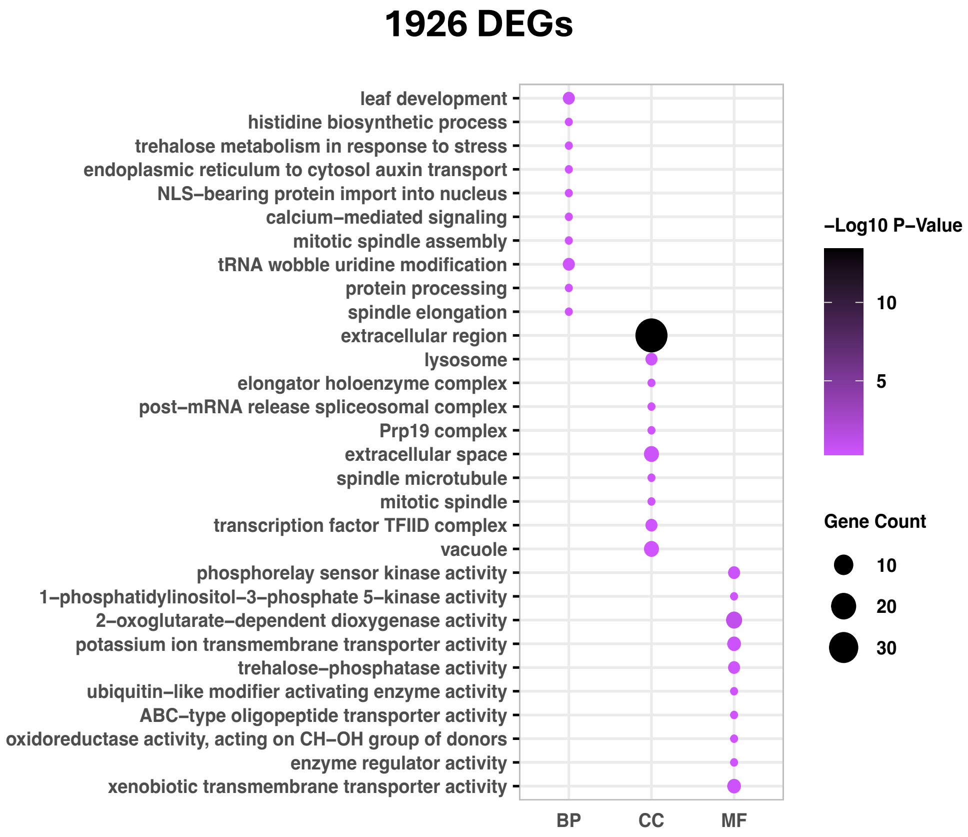

Suppelementary Figure S5 GO terms (BP, CC, and MF) detected in the 1,926 DEGs across three conditions except for WO97-N-4w.

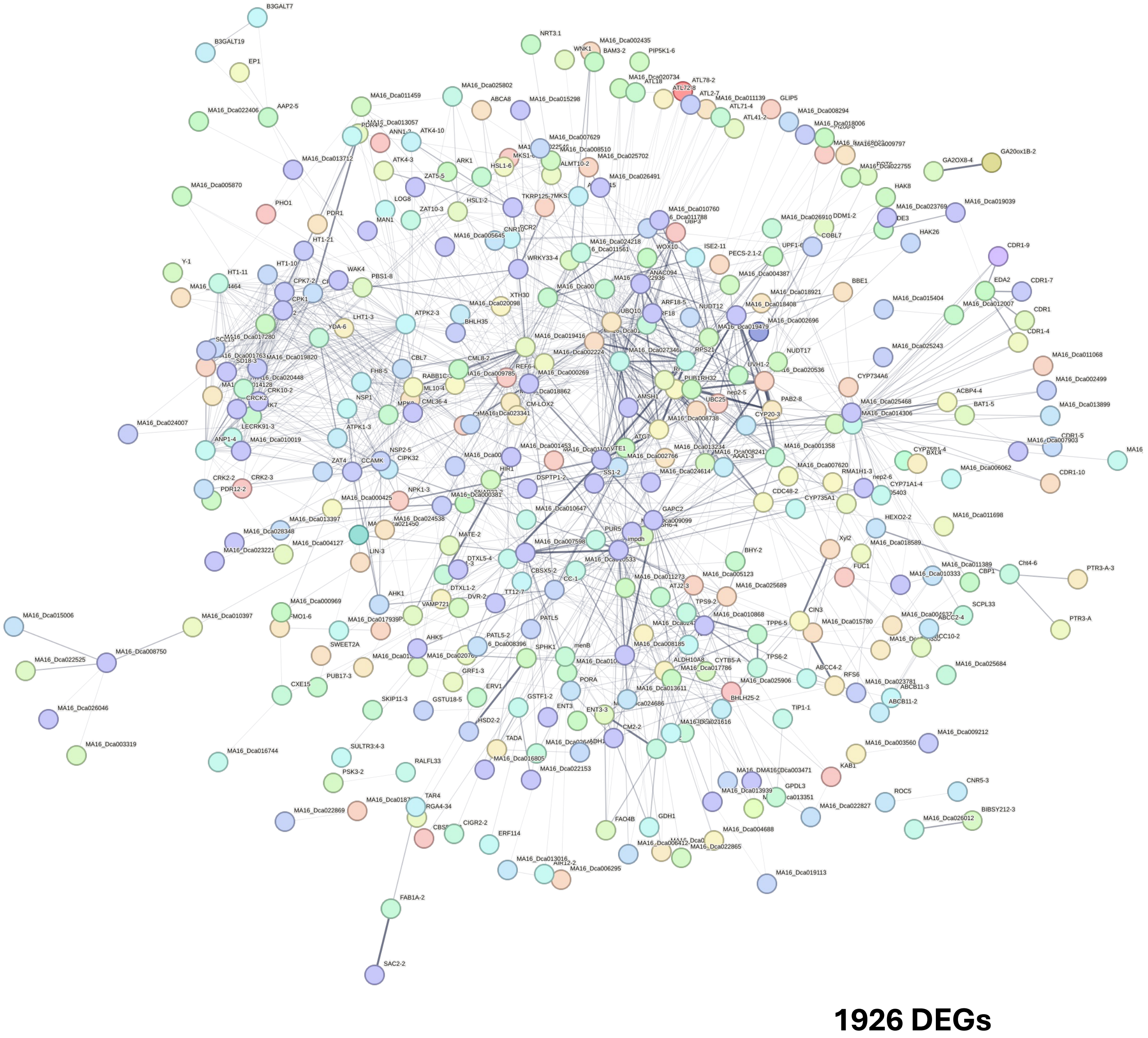

Suppelementary Figure S6 Protein-protein interaction (PPI) network constructed using 1,926 shared DEGs.

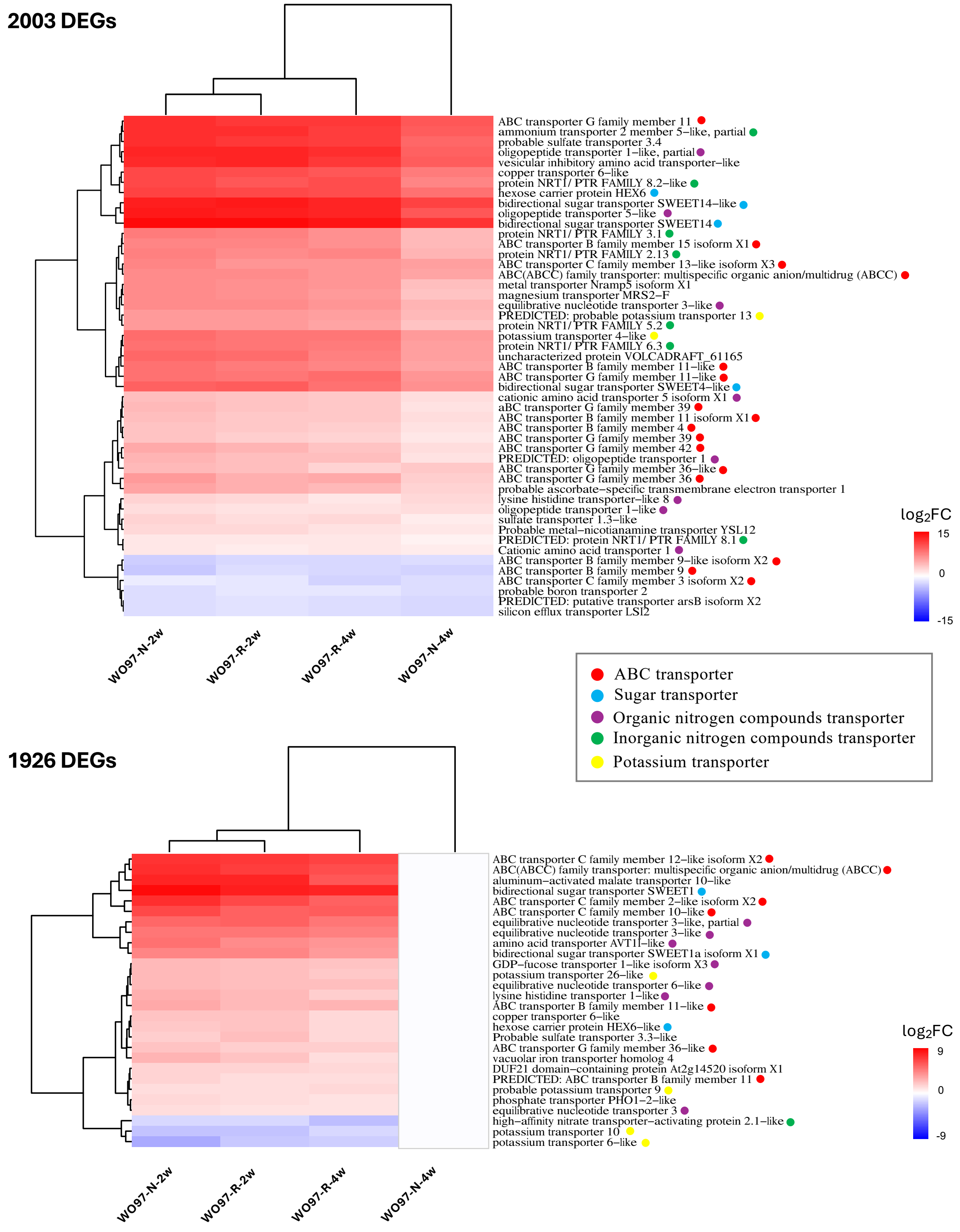

Suppelementary Figure S7 Heatmap reflecting the expression patterns for transporter genes among 2,003 and 1,926 shared DEGs. Heatmap reveals the expression patterns of transporter genes among 2,003 shared DEGs across all four tested symbiotic conditions (WO97-R -2w, WO97-N-2w, WO97-R-4w, and WO97-N-4w) and 1,926 shared DEGs across three symbiotic conditions, excluding WO97-N-4w. The heatmap was generated using Seaborn and Matplotlib in Python.
